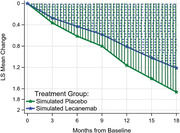# Application of restricted mean survival time method in Clinical Trials for Alzheimer disease

**DOI:** 10.1002/alz.087734

**Published:** 2025-01-03

**Authors:** Guogen Shan

**Affiliations:** ^1^ University of Florida, College of Public Health and Health Professions and College of Medicine, Gainesville, FL USA

## Abstract

**Background:**

The restricted mean disease progression (RMDP) is a measure of disease progression from baseline to a pre‐specified follow‐up visit. The RMST is a new statistical tool for survival analysis that is increasingly used in clinical trials. The RMST can be easily interpreted as the average survival time of patients from baseline to the pre‐specified follow‐up time.

**Method:**

We developed a new restricted mean disease progression (RMDP) scale to estimate time savings based on the RMST approach.

**Result:**

We found that the mean squared error (MSE) of the two new methods based on the RMDP is 30% less than that of the existing methods.

**Conclusion:**

The saved time of the two new approaches is more accurate than that of the exiting methods in many applications.